# Correlation Between Prognostic Nutritional Index and Heart Failure in Adults with Diabetes in the United States: Study Results from NHANES (1999–2016)

**DOI:** 10.31083/RCM25618

**Published:** 2025-01-20

**Authors:** Qiyuan Bai, Hao Chen, Zhen Gao, Xuhua Li, Jiapeng Li, Shidong Liu, Bing Song, Cuntao Yu

**Affiliations:** ^1^The First Clinical Medical College of Lanzhou University, 730000 Lanzhou, Gansu, China; ^2^Department of Cardiac Surgery, Capital Medical University Affiliated Beijing Anzhen Hospital, Beijing Institute of Heart Lung and Blood Vessel Diseases, 100029 Beijing, China; ^3^Department of Orthopaedics, Shanghai Sixth People's Hospital Affiliated to Shanghai Jiao Tong University School of Medicine, 200233 Shanghai, China; ^4^Institute of Microsurgery on Extremities, Shanghai Sixth People's Hospital Affiliated to Shanghai Jiao Tong University School of Medicine, 200233 Shanghai, China; ^5^Department of Cardiovascular Surgery, First Hospital of Lanzhou University, 730013 Lanzhou, Gansu, China; ^6^Department of Cardiovascular Surgery, Fuwai Hospital, National Center for Cardiovascular Diseases, Chinese Academy of Medical Sciences, Peking Union Medical College, 100006 Beijing, China

**Keywords:** prognostic nutritional index, diabetes, heart failure, NHANES

## Abstract

**Background:**

The relationship between diabetes and heart failure significantly impacts public health. This study assessed the prognostic nutritional index (PNI) as a predictor of heart failure risk in adult diabetic patients.

**Methods:**

An analysis was performed on 1823 diabetic adults using data collected from the National Health and Nutrition Examination Survey (NHANES) between 1999 and 2016. Serum albumin levels and lymphocyte counts were combined to calculate the PNI. We used descriptive statistics categorized by PNI quartiles and performed multivariate logistic regression to adjust for variables including age, gender, ethnicity, and coexisting medical conditions.

**Results:**

The median age (mean ± SD) was 59.942 ± 12.171 years, and the mean value ± SD of the PNI was 52.412 ± 5.430. The prevalence of heart failure was 7.405%. In the fully adjusted model, for each 1-unit increase in PNI, the risk of heart failure decreased by 8.2% (odds ratio (OR), 0.918; 95% confidence interval (CI) 0.884, 0.953). Participants in the highest PNI quartile (Q4) had a 63% reduced risk of heart failure compared to those in the lowest quartile (Q1). Tests for interactions did not reveal any statistically significant differences among these stratified subgroups (*p* for interaction > 0.05).

**Conclusions:**

This study demonstrated that a higher PNI was significantly associated with a decreased prevalence of heart failure in adults with diabetes.

## 1. Introduction

Diabetes, a prevalent chronic illness, is an endocrine condition defined by elevated blood glucose levels [1]. The main types include type 1 diabetes mellitus (T1DM), type 2 diabetes mellitus (T2DM), and gestational diabetes [2, 3], with T2DM accounting for over 90% of all cases [4]. Diabetes poses a significant threat to human health [5], as per data from the International Diabetes Federation (IDF), with approximately 537 million people worldwide receiving a diagnosis of diabetes in 2021. The projected estimate for 2030 is 643 million, and by 2045, it could increase to 783 million, thus potentially burdening global public health systems [6]. The numerous complications associated with diabetes, including cardiovascular diseases, retinopathy, kidney diseases, and neurological disorders, significantly increase the level of difficulty in patient care and the potential for diabetes-lined mortality [7]. Heart failure has become a significantly serious public health problem, and its incidence is increasing, resulting in considerable expenses related to hospitalization on a worldwide scale [8]. Research currently indicates a significant association between heart failure and diabetes, whereby patients diagnosed with diabetes are at a much greater risk of developing heart failure than non-diabetics. Moreover, there is a noticeable relationship between heart failure and an increase in the incidence of newly diagnosed diabetes [9]. Although the molecular mechanisms connecting these conditions have not been fully elucidated [10], it is evident that heart failure increases the mortality rate among diabetic patients, significantly affecting their quality of life and prognosis [11].

Buzby *et al*. [12] proposed the prognostic nutritional index (PNI), which utilizes serum albumin levels along with peripheral blood lymphocyte counts to evaluate an individual’s nutritional status and immune function [13]. The simplicity and objectivity of this method have led to a surge in research on the PNI, with it now commonly employed to evaluate preoperative nutritional status, postoperative outcomes, and levels of inflammation [14, 15]. Proper nutrition is essential in preventing and treating long-term health conditions such as diabetes [16]. Adequate dietary control and nutritional supervision may enhance the outlook for individuals with diabetes. A study has demonstrated that the PNI serves as a stand-alone predictor of unfavorable results in individuals with cardiac issues [17], and its prognostic precision exceeds that of albumin or lymphocyte counts. Therefore, PNI provides a new monitoring index for clinical research with potential clinical applications.

There is currently a paucity of research on heart failure and the PNI in diabetic adults. This research aimed to explore the relationship among these factors in adult individuals with diabetes by examining the National Health and Nutrition Examination Survey (NHANES) dataset. This study intended to assess the effectiveness of PNI as a predictive tool, providing clinical evidence that may help improve the prognosis and treatment management of diabetic patients.

## 2. Methods

### 2.1 Data Source and Participants

**Fig. 1.**
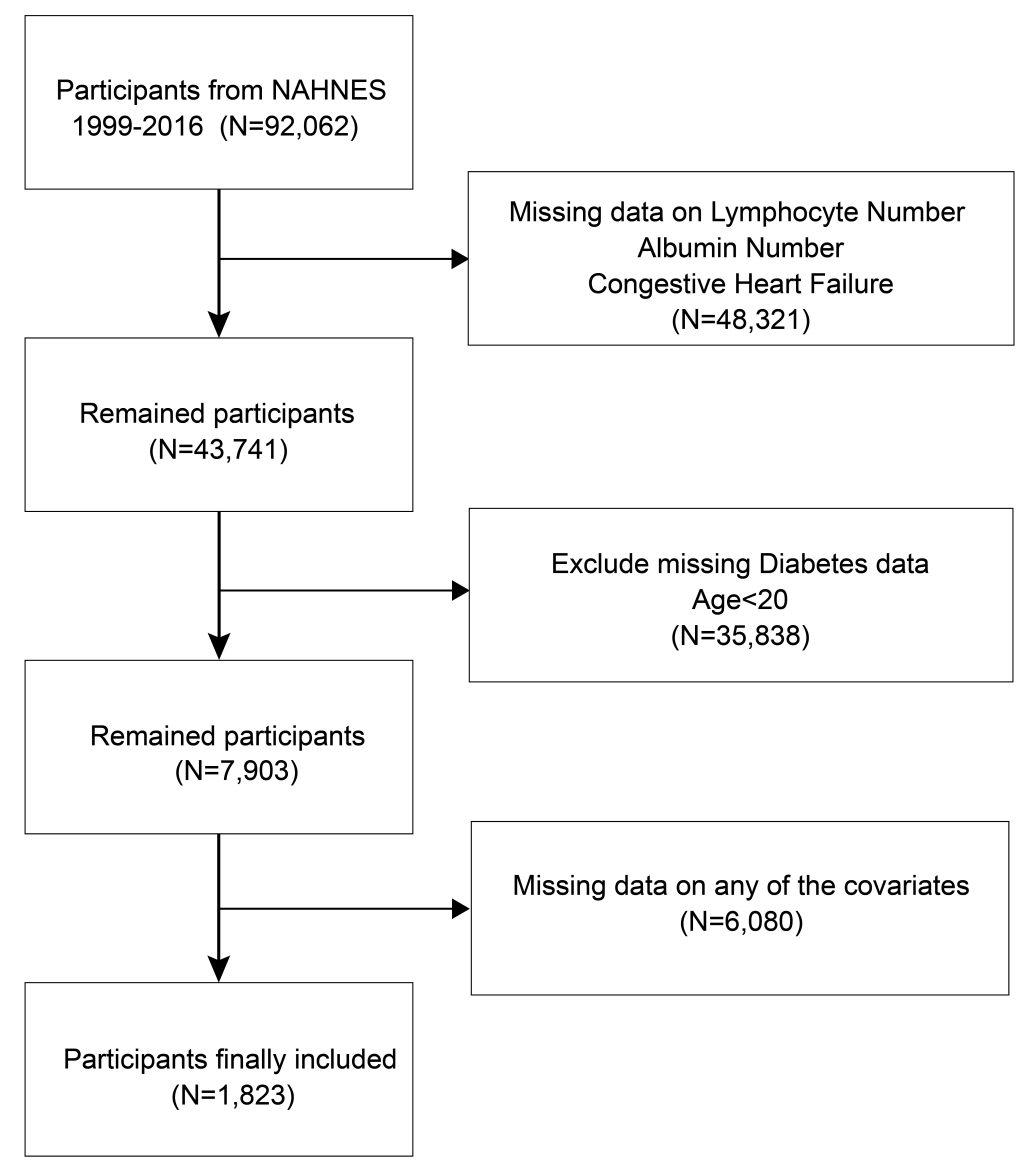
**Flowchart of the included participants**. NHANES, National Health and Nutrition Examination Survey.

The NHANES dataset used in this study was derived from a nationwide cross-sectional investigation by the National Center for Health Statistics (NCHS). A stratified multistage probability sampling technique was utilized to acquire a representative sample of the non-institutionalized civilian U.S. population. The NCHS Research Ethics Review Board approved the research activities, and all participants provided their informed consent prior to the study. The data collected included demographic information, physical exams, lab tests, health questionnaires, and prescribed medication records, all managed using a sophisticated computer system. This study analyzed the NHANES dataset from 1999 to 2016, which included 92,062 participants. In the data screening process, cases under the age of 20 and those with missing data on diabetes, PNI-related indicators, heart failure, and other relevant covariates were excluded. Ultimately, 1823 adults diagnosed with diabetes were included (Fig. 1). The diagnosis of diabetes was derived from questionnaires and laboratory tests. Participants were eligible if they answered affirmatively to any of the following inquiries: “Have you been diagnosed with diabetes by a medical professional?”, “Do you currently take insulin?”, or “Do you currently take medication for blood sugar control?”, or if their lab results met the diagnostic criteria for diabetes, which included glycated hemoglobin (%) ≥6.5% or a fasting blood glucose (mg/dL) ≥126 mg/dL. This approach integrated self-reported and biomarker information to improve the precision and dependability for the detection and diagnosis of diabetes.

### 2.2 Exposure and Outcomes

The PNI was utilized as the independent variable in this investigation, using the formula PNI = 5 × lymphocyte count (measured in units of 10^9^/L) + serum albumin levels (measured in units of g/L) [18]. Lymphocyte counts were derived from a complete blood count (CBC), utilizing Beckman Coulter technology to analyze cell count and size [19]. This technology is broadly acknowledged for assessing dietary nutritional status and immune function. Serum albumin levels, indicative of nutritional status, were measured utilizing the bromocresol purple dye-binding technique as documented in the NHANES database [20]. Higher PNI values typically indicate better nutritional status. The outcome variable was defined as heart failure. Data on heart failure in the NHANES study were mainly gathered via personal interviews that relied on self-reporting. This study used the “MCQ160B” variable from the NHANES questionnaire to diagnose heart failure. This variable specifically asks participants: “Has a doctor or other health professional ever told you that you have congestive heart failure?”. Participants who confirmed receiving information about their heart failure diagnosis from a healthcare professional (such as a doctor) were classified as having heart failure. Although the NHANES dataset lacks key diagnostic markers (such as B-type natriuretic peptide (BNP), N-terminal pro B-type natriuretic peptide (NT-proBNP), troponin, electrocardiograms (EKGs), and cardiac imaging), and reliance on questionnaire information may introduce some ambiguity, previous studies have shown that self-reported data are valid for diagnosing heart failure among NHANES participants [21, 22, 23]. Across different racial and age groups, self-reported data have previously effectively described overall trends and racial differences [24]. Additionally, A study has shown that while self-reported heart failure data may have lower sensitivity, they exhibit high specificity (96–97%) and have significant application value in large-scale epidemiological studies [25].

### 2.3 Covariables

This study examined various covariates, encompassing demographic information including gender, age, race, marital status, educational attainment, and income level. In addition, it integrated medical history co-morbidities such as hypertension, stroke, coronary heart disease, angina, and myocardial infarction. Specific survey questionnaires were used to collect lifestyle characteristics; within this framework, individuals classified as smokers were those who had consumed a minimum of 100 cigarettes throughout their lifetime [26]. Similarly, individuals who had consumed alcohol on at least 12 distinct occasions were classified as drinkers [27]. NHANES grouped race and ethnicity by the responses provided to survey questions. Categories included Mexican American, non-Hispanic white, non-Hispanic black, and other ethnicities. Marital status was divided into two groups: unmarried (comprising never married, divorced, separated, and widowed) and married (including married and cohabiting individuals). Education level was categorized as less than high school, high school, and more than high school.

Blood pressure and body mass index (BMI) were assessed through laboratory examinations, with BMI divided into three groups: normal (BMI ranging from 18.5 to 25 kg/m^2^), overweight (BMI between 25 and 30 kg/m^2^), and obese (BMI exceeding 30 kg/m^2^) [28].

Details and information regarding these covariates can be accessed on the official website of the Centers for Disease Control and Prevention at https://www.cdc.gov/nchs/nhanes/.

### 2.4 Missing Covariables

In handling missing covariables in the study data, this research adopted the strategy of directly deleting samples containing missing data [29]. This approach offers several significant advantages. First, it avoids the potential biases introduced by data imputation, thus ensuring the accuracy and reliability of the analysis results. Second, this method simplifies the data processing workflow, eliminating the need for complex statistical imputation techniques and thereby enhancing the robustness of the study findings. Moreover, removing samples with missing data reduces the errors that incorrect data imputation could cause, making the study results more credible. This approach is particularly helpful in maintaining the overall quality of the research, especially when addressing issues with missing data, which are difficult to interpret.

### 2.5 Statistical Analysis

All data analyses followed the protocols outlined by the Centers for Disease Control and Prevention (CDC), available at https://wwwn.cdc.gov/nchs/nhanes/tutorials/default.aspx. The research included descriptive analyses of the data from all participants. Percentages were used to represent categorical variables, while distribution properties detailed continuous variables. This analysis was conducted using either the mean and standard deviation (SD) or the median and interquartile range (IQR) [30].

Continuous variables were analyzed using the Student’s *t*-test to evaluate differences in clinical features, and categorical variables were assessed with the chi-square test. This approach identified notable differences among variables, providing a methodologically sound basis for subsequent analysis. This study used logistic regression models with weights to assess the relationship between the continuous PNI and its quartiles, alongside the risk of developing heart failure. The study calculated odds ratios (ORs) and 95% confidence intervals (CIs) using three distinct regression models: The initial model, Model 1, incorporated only the PNI variable. Model 2 accounted for additional variables such as gender, age (as a continuous variable), race (classified as Mexican American, non-Hispanic white, non-Hispanic black, and other), and body mass index (BMI categorized as normal (18.5 < BMI < 25 kg/m^2^), overweight (25 ≤ BMI ≤ 30 kg/m^2^), and obese (BMI > 30 kg/m^2^)). Model 3 included further refinements to adjust for education level (less than high school, high school, more than high school), marital status (unmarried, married), income relative to the poverty threshold (continuous variable), smoking status (yes, no), alcohol consumption status (yes, no), and cardiovascular conditions, such as stroke, coronary heart disease, angina, myocardial infarction, and hypertension.

Analyses were conducted on subgroups to investigate differences among variables, including gender, race, marital status, level of education, BMI, smoking habits, alcohol consumption, and cardiovascular health. These analyses employed weighted stratified linear regression models to test for interaction terms between subgroups, evaluating differences in effects. Significant statistical results were identified through the study of statistics, with a *p*-value less than 0.05.

Analysis was conducted using R version 3.4.3 (found at http://www.R-project.org, given by The R Foundation), Empower program (found at http://www.empowerstats.com/, created by X&Y Solutions, Inc., Boston, MA, USA), and DecisionLinnc 1.0 program (found at https://www.statsape.com/). DecisionLinnc is a system that combines several coding environments, making it easier to handle data, conduct analyses, and utilize machine learning through a graphical interface.

## 3. Results

### 3.1 Baseline Characteristics of Participants

**Fig. 2.**
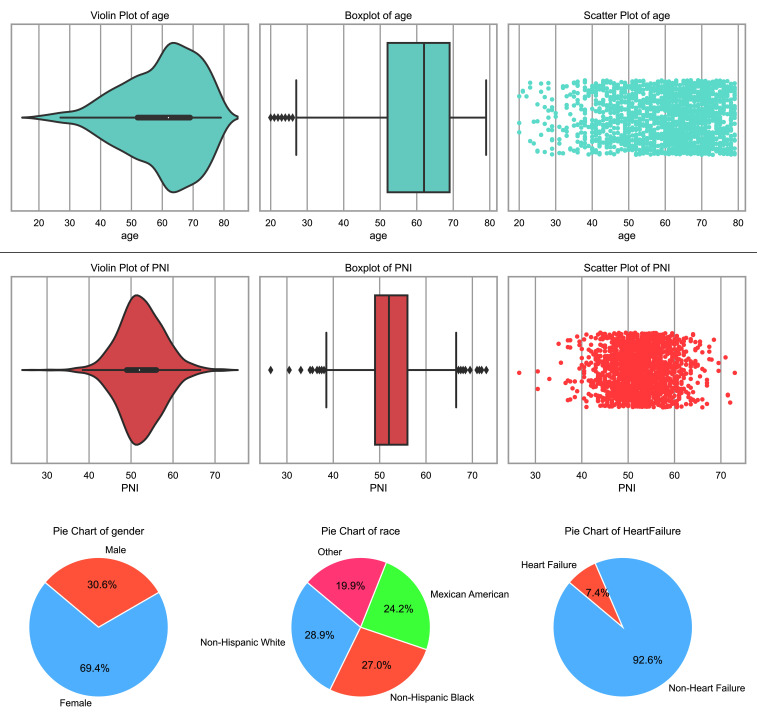
**Partial baseline characteristics and incidence of heart failure**. PNI, prognostic nutritional index.

This study included 1823 adults with diabetes, all meeting specific inclusion and exclusion criteria. The mean age of the participants was 59.94 years, with a standard deviation of 12.17 years. Among the participants, 30.55 were males, and 69.45% were females. The ethnical composition of the participants was diverse, with 28.85% identified as non-Hispanic whites, 27.04% as non-Hispanic blacks, 24.19% as Mexican Americans, and 19.91% belonging to other races. The mean PNI among the participants was 52.41, with a standard deviation of 5.43. Additionally, 7.41% of the subjects had heart failure, according to the data presented in Fig. 2.

Based on the PNI quartiles, the clinical characteristics of participants were compared across different factors such as age, race, marital status, BMI, hypertension, myocardial infarction, and heart failure. Significant differences were found in these factors across the PNI quartiles, with *p*-values less than 0.05 (Table 1). Participants in the Q4 group were generally younger, more likely to be Mexican American, married or cohabiting, had a BMI between 18.5 and 30 kg/m^2^, and were less likely to have hypertension, myocardial infarction, or heart failure than those in the Q1 group. These findings suggest that there may be correlations between the PNI quartiles and certain clinical characteristics of the participants. Overall, the results indicate notable disparities in age, race, marital status, BMI, and various cardiovascular health conditions across different PNI quartiles. Participants in the Q4 group exhibited specific characteristics such as being younger, Mexican American, married or cohabiting, and having a healthier BMI range, along with lower rates of hypertension, myocardial infarction, and heart failure. These findings shed light on the potential associations between the PNI quartiles and the clinical profiles of individuals participating in the study. Additional research could further explore these relationships to improve understanding of the implications for healthcare interventions and strategies targeting specific demographic groups.

**Table 1.** **Baseline characteristics of participants between 1999 and 2016 (N = 1823)**.

PNI		Q1	Q2	Q3	Q4	*p*-value
		N = 419	N = 432	N = 500	N = 472	
Age, (years)		61.06 ± 11.81	61.11 ± 11.72	59.97 ± 12.16	57.85 ± 12.65	<0.001
Gender, (%)						0.347
	Male	115 (27.446%)	142 (32.870%)	158 (31.600%)	142 (30.085%)	
	Female	304 (72.554%)	290 (67.130%)	342 (68.400%)	330 (69.915%)	
Race, (%)						<0.001
	Mexican American	81 (19.332%)	106 (24.537%)	124 (24.800%)	130 (27.542%)	
	Non-Hispanic White	124 (29.594%)	142 (32.870%)	135 (27.000%)	125 (26.483%)	
	Non-Hispanic Black	147 (35.084%)	116 (26.852%)	122 (24.400%)	108 (22.881%)	
	Other	67 (15.990%)	68 (15.741%)	119 (23.800%)	109 (23.093%)	
Education, (%)						0.226
	Less than high school	173 (41.289%)	166 (38.426%)	212 (42.400%)	223 (47.246%)	
	High school or GED	90 (21.480%)	104 (24.074%)	114 (22.800%)	98 (20.763%)	
	Above high school	156 (37.232%)	162 (37.500%)	174 (34.800%)	151 (31.992%)	
Marital status, (%)						0.029
	Unmarried	196 (46.778%)	181 (41.898%)	206 (41.200%)	174 (36.864%)	
	Married	223 (53.222%)	251 (58.102%)	294 (58.800%)	298 (63.136%)	
BMI, (kg/m^2^)						<0.001
	<25	39 (9.308%)	47 (10.880%)	75 (15.000%)	72 (15.254%)	
	25–30	89 (21.241%)	126 (29.167%)	131 (26.200%)	138 (29.237%)	
	>30	291 (69.451%)	259 (59.954%)	294 (58.800%)	262 (55.508%)	
Smoker, (%)						0.321
	Yes	143 (34.129%)	128 (29.630%)	163 (32.600%)	166 (35.169%)	
	No	276 (65.871%)	304 (70.370%)	337 (67.400%)	306 (64.831%)	
Alcohol use, (%)						0.701
	Yes	208 (49.642%)	219 (50.694%)	267 (53.400%)	244 (51.695%)	
	No	211 (50.358%)	213 (49.306%)	233 (46.600%)	228 (48.305%)	
Hypertension, (%)						0.035
	Yes	329 (78.520%)	330 (76.389%)	357 (71.400%)	339 (71.822%)	
	No	90 (21.480%)	102 (23.611%)	143 (28.600%)	133 (28.178%)	
Stroke, (%)						0.628
	Yes	34 (8.115%)	34 (7.870%)	40 (8.000%)	29 (6.144%)	
	No	385 (91.885%)	398 (92.130%)	460 (92.000%)	443 (93.856%)	
CHD, (%)						0.114
	Yes	44 (10.501%)	45 (10.417%)	39 (7.800%)	32 (6.780%)	
	No	375 (89.499%)	387 (89.583%)	461 (92.200%)	440 (93.220%)	
Angina, (%)						0.079
	Yes	39 (9.308%)	28 (6.481%)	29 (5.800%)	25 (5.297%)	
	No	380 (90.692%)	404 (93.519%)	471 (94.200%)	447 (94.703%)	
Myocardial infarction, (%)						<0.001
	Yes	57 (13.604%)	34 (7.870%)	37 (7.400%)	21 (4.449%)	
	No	362 (86.396%)	398 (92.130%)	463 (92.600%)	451 (95.551%)	
Family PIR		2.043 ± 1.438	2.049 ± 1.422	2.064 ± 1.471	2.013 ± 1.413	0.992
Lymphocyte, (10^9^/L)		1.562 ± 0.480	1.922 ± 0.436	2.316 ± 0.524	3.086 ± 0.729	<0.001
Albumin, (g/L)		37.675 ± 3.345	40.725 ± 2.163	42.054 ± 2.518	43.744 ± 2.832	<0.001
Heart failure, (%)						<0.001
	Yes	59 (14.081%)	29 (6.713%)	29 (5.800%)	18 (3.814%)	
	No	360 (85.919%)	403 (93.287%)	471 (94.200%)	454 (96.186%)	

Continuous variables are presented as the mean ± SD. The *p*-value was calculated using the weighted linear regression model. (%) was applied for the categorical variables, and the *p*-value was calculated using the weighted chi-square test. Abbreviation: PNI, prognostic nutritional index; CHD, coronary heart disease; BMI, body mass index; Family PIR, the ratio of family income to poverty; GED, General Educational Development.

### 3.2 Association between PNI and Heart Failure 

Table 2 illustrates the results of the multivariable regression analysis evaluating the link between PNI and the risk of heart failure across three models, each with distinct levels of adjustment. Across all models, PNI exhibited a notable negative correlation with the risk of heart failure. The OR for PNI in the unadjusted Model 1 was 0.900 (95% CI: 0.871–0.930). For the preliminarily adjusted Model 2, the OR was 0.908 (95% CI: 0.877–0.940), and for the fully adjusted Model 3, the OR was 0.918 (95% CI: 0.884–0.953). This indicates that as PNI increases, the risk of developing heart failure progressively declines.

**Table 2.** **The association of PNI and heart failure**.

Exposure		OR (95% CI)		
		Model 1	Model 2	Model 3
		(N = 1823)	(N = 1823)	(N = 1823)
PNI		0.90 (0.98, 0.93)	0.91 (0.88, 0.94)	0.92 (0.88, 0.95)
		<0.001	<0.001	<0.001
Quartiles				
	Quartile 1	1.0	1.0	1.0
	Quartile 2	0.44 (0.28, 0.70)	0.46 (0.29, 0.74)	0.54 (0.32, 0.91)
	Quartile 3	0.38 (0.24, 0.60)	0.42 (0.26, 0.68)	0.48 (0.29, 0.81)
	Quartile 4	0.24 (0.14, 0.42)	0.30 (0.17, 0.53)	0.37 (0.20, 0.68)
*p* for trend		<0.001	<0.001	<0.001

The sensitivity analysis converted PNI from a continuous variable to a categorical variable (quartile). Model 1: No covariates were adjusted. Model 2: Age, gender, race/ethnicity, and BMI were adjusted. Model 3: Age, gender, race/ethnicity, BMI, educational level, marital status, family PIR, smoking status, drinking status, stroke status, CHD status, hypertension status, angina status, and myocardial infarction status were adjusted. Abbreviation: OR, odds ratio; 95% CI, 95% confidence interval; PNI, prognostic nutritional index; CHD, coronary heart disease; BMI, body mass index; family PIR, the ratio of family income to poverty.

Additional analysis of the PNI quartiles further confirms this pattern. When considering all variables in Model 3, individuals in the lowest quartile (Q1, OR = 1.00) had a higher risk of heart failure compared to those in the second quartile (Q2, OR = 0.538, 95% CI: 0.319–0.909), third quartile (Q3, OR = 0.480, 95% CI: 0.285–0.808), and fourth quartile (Q4, OR = 0.370, 95% CI: 0.202–0.676), all of whom showed a significantly decreased risk of heart failure. Particularly in the fourth quartile, compared to the baseline, the risk of heart failure was reduced by 63.0%, demonstrating strong statistical significance (*p* trend <0.001), highlighting the clear association between higher PNI values and the reduced risk of heart failure (Table 2).

**Fig. 3.**
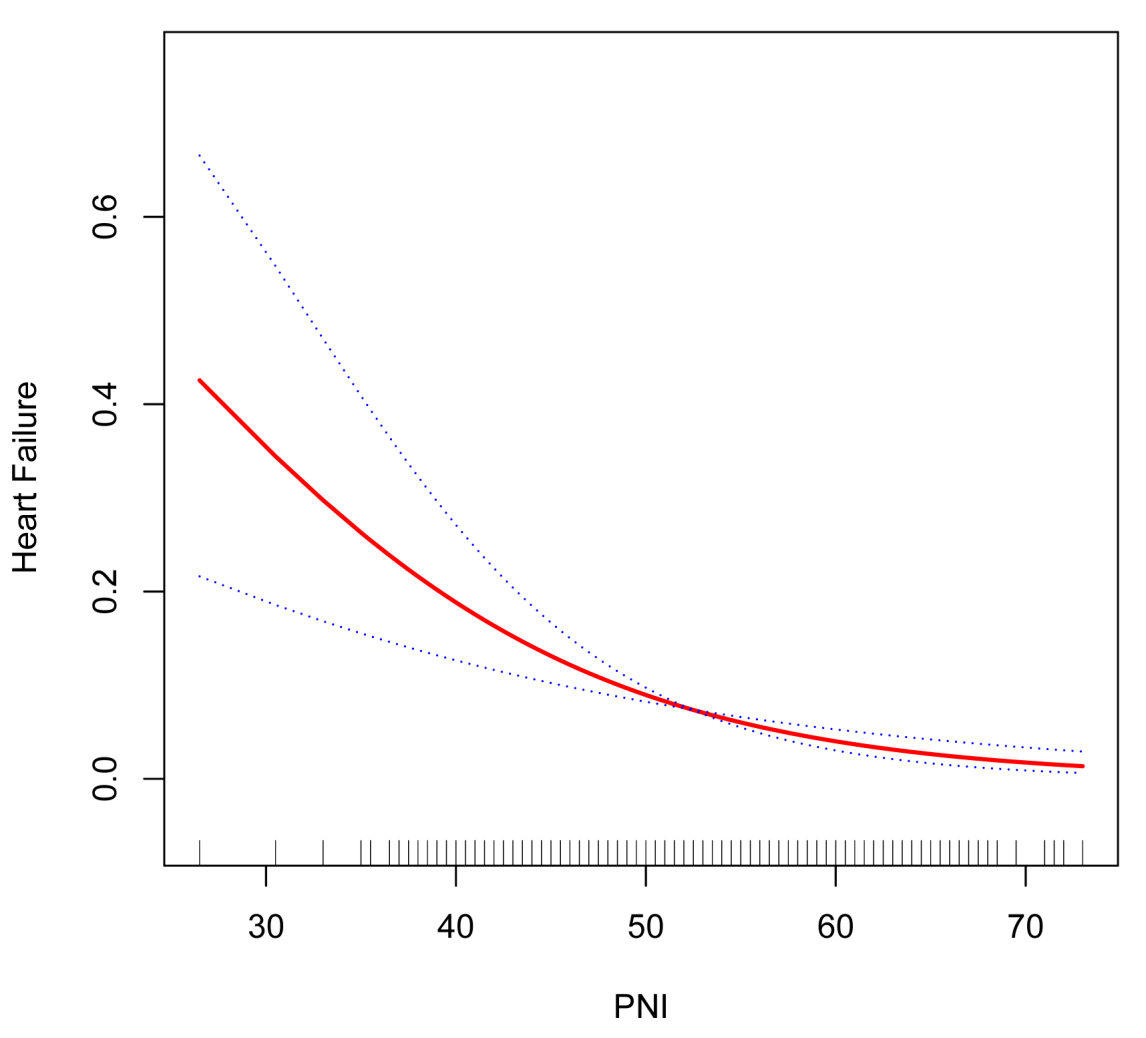
**Relationship between PNI and heart failure**. PNI, prognostic nutritional index.

Additionally, we explored the relationship between PNI and heart failure status using a smoothing curve fitting method. The results showed a clear nonlinear negative correlation between the two, as depicted in Fig. 3. This underscores the complex association pattern between PNI and the risk of heart failure, revealing dynamic trends in their relationship.

### 3.3 Subgroup Analysis 

**Fig. 4.**
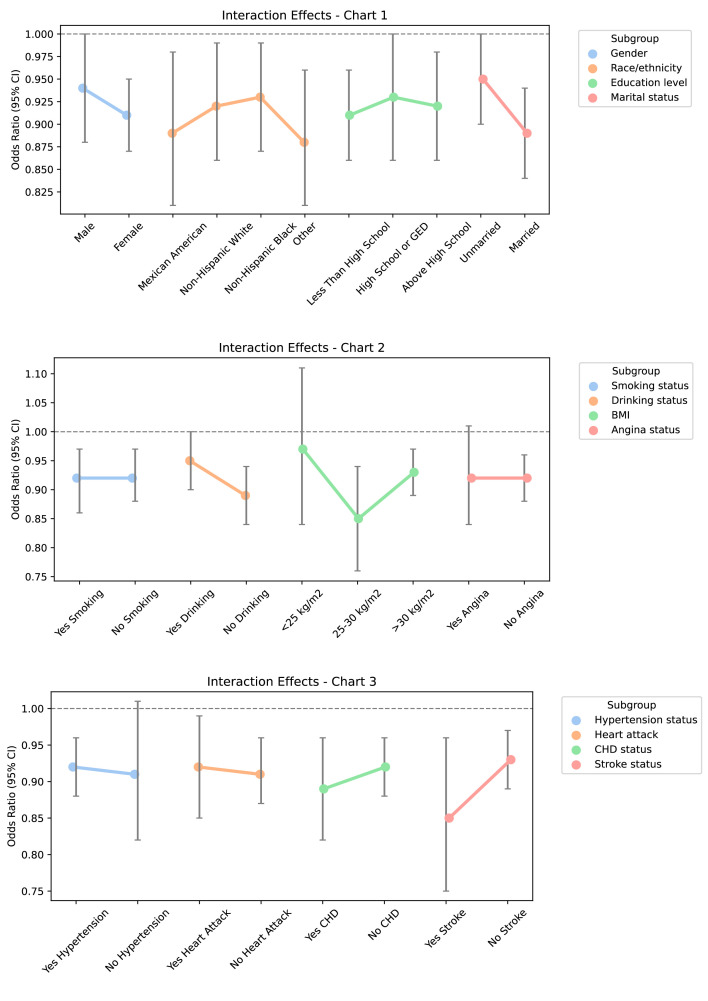
**Subgroup analysis and interaction analysis**. Abbreviation: GED, General Educational Development; CHD, coronary heart disease; BMI, body mass index; 95% CI, 95% confidence interval.

Subgroup analyses were performed to evaluate the consistency of the relationship between PNI and heart failure across various demographic groups. As shown in Table 3, the analysis results indicate that the correlation between PNI and heart failure was inconsistent across different subgroups. In particular, significant associations were noted in subgroups categorized by ethnicity, marital status, tobacco use, alcohol consumption, cerebrovascular accident, coronary artery disease, and myocardial infarction (*p* < 0.05). However, tests for interactions did not reveal any statistically significant differences among these stratified subgroups. This indicates that factors such as gender, race, educational level, marital status, smoking status, alcohol consumption, BMI, hypertension, history of stroke, coronary heart disease, angina, and myocardial infarction do not influence the inverse relationship between PNI and heart failure risk (*p* for interaction > 0.05) (Fig. 4). This emphasizes the universality of the relationship between higher PNI and reduced risk of heart failure, indicating certain stability and broad applicability of this link across different subgroups.

**Table 3.** **Results of subgroup analysis and interaction analysis**.

Subgroup		OR (95% CI)	*p*-value	*p* for interaction
Gender				0.492
	Male	0.94 (0.88, 1.00)	0.055	
	Female	0.91 (0.87, 0.95)	<0.001	
Race/ethnicity				0.757
	Mexican American	0.89 (0.81, 0.98)	0.013	
	Non-Hispanic White	0.92 (0.86, 0.99)	0.019	
	Non-Hispanic Black	0.93 (0.87, 0.99)	0.028	
	Other	0.88 (0.81, 0.96)	0.005	
Education level				0.889
	Less than high school	0.91 (0.86, 0.96)	0.001	
	High school or GED	0.93 (0.86, 1.00)	0.064	
	Above high school	0.92 (0.86, 0.98)	0.015	
Marital status				0.065
	Unmarried	0.95 (0.90, 1.00)	0.050	
	Married	0.89 (0.84, 0.94)	<0.001	
Smoking status				0.896
	Yes	0.92 (0.86, 0.97)	0.003	
	No	0.92 (0.88, 0.97)	0.001	
Drinking status				0.090
	Yes	0.95 (0.90, 1.00)	0.041	
	No	0.89 (0.84, 0.94)	<0.001	
BMI				0.205
	<25 kg/m^2^	0.97 (0.84, 1.11)	0.616	
	25–30 kg/m^2^	0.85 (0.76, 0.94)	0.002	
	>30 kg/m^2^	0.93 (0.89, 0.97)	0.001	
Angina status		1.17 (0.88, 1.56)	0.275	0.981
	Yes	0.92 (0.84, 1.01)	0.080	
	No	0.92 (0.88, 0.96)	<0.001	
Hypertension status				0.826
	Yes	0.92 (0.88, 0.96)	<0.001	
	No	0.91 (0.82, 1.01)	0.066	
Myocardial infarction				0.931
	Yes	0.92 (0.85, 0.99)	0.023	
	No	0.91 (0.87, 0.96)	<0.001	
CHD status				0.411
	Yes	0.89 (0.82, 0.96)	0.004	
	No	0.92 (0.88, 0.96)	0.003	
Stroke status				0.1562
	Yes	0.85 (0.75, 0.96)	0.007	
	No	0.93 (0.89, 0.97)	<0.001	

Abbreviation: OR, odds ratio; 95% CI, 95% confidence interval; CHD, coronary heart disease; BMI, body mass index; GED, General Educational Development.

## 4. Discussion

Based on the NHANES public database, this study is the first to clearly demonstrate a negative correlation between the PNI and heart failure incidence among adults with diabetes. Using three models to gradually adjust for confounding factors yielded consistent results, indicating that a higher PNI is associated with a reduced risk of heart failure, with statistically significant differences. The coexistence of diabetes and heart failure poses a serious clinical challenge [31]. Patients with diabetes are in a state of chronic hyperglycemia, which leads to metabolic disturbances in cardiac cells and directly damages cardiomyocytes [32, 33]. Additionally, microvascular disease, structural and functional changes in the myocardium, and the development of atherosclerosis further increase the complexity and risk of heart failure [34, 35]. No studies have directly explored the relationship between these two factors in the diabetic population. This finding supports the potential of PNI as an effective biomarker for heart failure in diabetic patients, suggesting that PNI may become a valuable clinical tool for assessing the risk of heart failure.

The results of this study are consistent with previous research on the relationship between PNI and heart failure prognosis. Zhang *et al*. [36] followed 1048 patients with metabolic syndrome and heart failure, and their findings indicated that the PNI is an independent predictor of all-cause mortality and cardiovascular death in these patients, with a negative correlation between the PNI and adverse outcomes. In older patients with heart failure, low PNI values have also been associated with both short and long-term mortality [37]. Kawata *et al*. [38] explored the relationship between changes in PNI during hospitalization and outcomes in patients with acute heart failure, concluding that higher PNI levels are independently associated with better outcomes in heart failure patients. Other studies have also confirmed similar conclusions [39, 40].

PNI reflects an individual’s nutritional status [41, 42] and immune function [43, 44]. Malnutrition can lead to hypoproteinemia and weakened immunity, which in turn can trigger heart failure. Moreover, chronic hypoperfusion, congestion, and inflammatory responses in heart failure patients can impair liver and kidney function, leading to reduced albumin production and exacerbating malnutrition [45, 46, 47]. Inflammation activation and immune infiltration play critical roles in the pathological process of heart failure [48, 49]. Abnormal immune function can promote the progression of heart failure [50], and anti-myocardial autoreactivity by the adaptive immune system has been implicated in structural remodeling, functional decline, and the development of heart failure [51]. Individuals with good nutritional status typically possess strong metabolic capacity and immune responses, which are crucial for defending against infections and other stressors that may lead to heart failure [52]. Additionally, individuals with higher PNI levels generally have better overall health, healthier BMIs, and a lower likelihood of developing hypertension, myocardial infarction, and heart failure. Conversely, individuals with lower PNIs may face an increased risk of heart failure due to the combined effects of these factors.

This study is the first to focus on the diabetic population, exploring the relationship between PNI and heart failure and confirming that PNI remains negatively correlated with heart failure in this high-risk group. Although previous studies have demonstrated the significant value of using the PNI in general heart failure patients, caution is needed when directly extrapolating these findings to the general population, given the specific metabolic and pathophysiological characteristics of diabetic patients.

## 5. Limitations

This study has several limitations. First, due to the observational design of the study, the causality between the PNI and heart failure risk cannot be definitively established. Second, despite the adjustment for numerous confounding variables, there remains a possibility of undisclosed confounders that might influence the outcomes. Furthermore, the research predominantly relies on a sole assessment of PNI, failing to investigate how alterations in PNI levels over time could impact the likelihood of developing heart failure.

## 6. Conclusions

This research proposes a significant inverse relationship between the PNI and the risk of heart failure. This finding implies that PNI could be a valuable predictor of heart failure. However, further research is necessary to validate these results and to evaluate the influence of time-related variations in PNI on the occurrence of heart failure. Future research should also investigate the potential biological mechanisms that underlie the association between PNI and heart failure, aiming to enhance our understanding of these mechanisms for use in clinical practice.

## Availability of Data and Materials

Publicly available datasets were analyzed in this study. This data can be found here: https://www.cdc.gov/nchs/nhanes/index.htm.

## Supplementary Material

Supplementary material associated with this article can be found, in the online version, at https://doi.org/10.31083/RCM25618.
