# Supplementary material for: Correlation Between Prognostic Nutritional Index and Heart Failure in Adults with Diabetes in the United States: Study Results from NHANES (1999–2016)
Source: Rev Cardiovasc Med. 2025 Jan 20;26(1):25618. doi: 10.31083/RCM25618 (PMC11760548; doi:10.31083/RCM25618)
Supplement: Supplementary file 1 [file 2153-8174-26-1-25618-s1.docx]

**Summary of Studies on Prognostic Nutritional Index (PNI) in Heart Failure and Related Conditions**

| Author | Year | Title | Conclusion |
| --- | --- | --- | --- |
| Zhang X | 2023 | Prognostic Nutritional Index (PNI) as a Predictor in Patients with Metabolic Syndrome and Heart Failure | In patients with metabolic syndrome and heart failure, malnutrition assessed by PNI is an independent predictor for all-cause death and cardiovascular death, and PNI is negatively correlated with the occurrence of adverse outcomes. |
| Candeloro M | 2020 | Prognostic nutritional index in elderly patients hospitalized for acute heart failure. ESC Heart Fail | Low PNI values are associated with short-term and long-term mortality among elderly patients hospitalized for acute decompensated heart failure. |
| Kawata | 2022 | Changes in prognostic nutritional index during hospitalization and outcomes in patients with acute heart failure. Heart Vessels | In conclusion, changes in nutritional status during hospitalization, evaluated using the PNI on admission and at discharge, were independently associated with 1-year outcomes in patients with acute HF. |
| Ju C | 2021 | Derivation of an electronic frailty index for predicting short-term mortality in heart failure: a machine learning approach | The electronic frailty index based on co-morbidities, inflammation, and nutrition information can readily predict mortality outcomes. Their predictive performances were significantly improved by gradient boosting techniques. |
| Çinier G | 2021 | Prognostic nutritional index as the predictor of long-term mortality among HFrEF patients with ICD | Among patients who were implanted with ICD secondary to HFrEF, lower PNI value predicted all-cause mortality during long-term follow-up. |
| Our study | 2024 | Correlation Between Prognostic Nutritional Index and Heart Failure in Adults with Diabetes in the United States: Study Results from NHANES (1999–2016) | In the diabetic population, there is a negative correlation between the Prognostic Nutritional Index (PNI) and the incidence of heart failure among adults with diabetes. |

Abbreviation: PNI, Prognostic Nutritional Index; HFrEF, heart failure with reduced ejection fraction; ICD, implantable cardioverter defibrillator; NHANES, National Health and Nutrition Examination Survey.
